# Hyperuricemia and its association with adiposity and dyslipidemia in Northwest China: results from cardiovascular risk survey in Xinjiang (CRS 2008–2012)

**DOI:** 10.1186/s12944-020-01211-z

**Published:** 2020-04-01

**Authors:** Fen Liu, Guo-Li Du, Ning Song, Yi-Tong Ma, Xiao-Mei Li, Xiao-Ming Gao, Yi-Ning Yang

**Affiliations:** 1 State Key Laboratory of Pathogenesis, Prevention and Treatment of High Incidence Diseases in Central Asia, Clinical Medical Research Institute, the First Affiliated Hospital of Xinjiang Medical University, 137 Liyushan South Road, Urumqi, 830054 Xinjiang China; 2 Department of Cardiology, the First Affiliated Hospital of Xinjiang Medical University, Urumqi, China; 3 Department of Endocrinology, First Affiliated Hospital of Xinjiang Medical University, Urumqi, China; 4 Xinjiang Key Laboratory of Medical Animal Model Research, Urumqi, China

**Keywords:** Hyperuricemia, Adiposity, Dyslipidemia, Northwest China

## Abstract

**Background:**

Hyperuricemia predisposes to gout, which may result in tophi, kidney stones, or urate nephropathy even kidney failure. Many metabolic risk factors and disorders has been recognized as a key risk factor contributing to development of hyperuricemia.

**Aim:**

To determine the prevalence of hyperuricemia and its association with adiposity and dyslipidemia.

**Methods:**

We recruited non-hospitalized participants (aged ≥35 years) in Xinjiang, a northwest part of China based on the Cardiovascular Risk Survey (CRS 2008–2012). Information of general health status, seafood or internal organs intake and history of disease were obtained by using an interview-based questionnaire. The levels of serum uric acid (sUA) and creatinine and lipid profiles were measured. A multivariate logistic regression model was performed to assess the association between prevalence of hyperuricemia and adiposity and dyslipidemia.

**Results:**

This study recruited 16,611 participants, and 14,618 was included (mean age of 50.5 ± 12.6 years, 46.6% was males). The study population comprised three ethnic groups with 39.4% of Han, 32.6% of Uygur and 28% of Kazakh Chinese. The overall prevalence of hyperuricemia was 9.1% (95% CI: 8.6 to 9.6) and it was11.8% in men was 6.7% in women. The three ethnic groups also had different hyperuricemia prevalence with 15.4% in Han, 4.6% in Uygur and 5.5% in Kazakh Chinese, which corresponding to a respective mean sUA levels of 306.2 ± 86.9, 249.4 ± 76.1 and 259.8 ± 78.7 μmol/L. Participants with diabetes, hypertension or hypertriglyceridemia and higher blood urea nitrogen (BUN), estimated glomerular filtration rate (eGFR), fasting blood glucose (FBG), triglycerides (TG), total cholesterol (TC) had higher levels of sUA (*P* < 0.001 respectively). Multivariate logistic regression analysis revealed that age, gender, ethnicity, drinking, obesity, waist circumference, TG (≥2.26 mmol/L), TC (≥6.22 mmol/L) are major risk factors for hyperuricemia. Compared to the 35–44-year age group [adjusted odds ratio (AOR) = 1], the risk of hyperuricemia increased 1.61-fold in the 65–74-year age group (AOR = 1.61, 95% CI: 1.34–1.91; *P* < 0.001), and 1.71-fold in the 75- and older age group (AOR = 1.71, 95% CI: 1.27–2.29; *P* < 0.001). There was a 1.45-fold higher risk of hyperuricemia in men (AOR = 1.45, 95% CI: 1.24–1.68; *P* < 0.001) compared to women. Further, the risk of hyperuricemia increased significantly with drinking (AOR = 1.36; 95% CI: 1.16–1.61; *P* < 0.001), overweight (AOR = 1.25; 95% CI: 1.06–1.48; *P* = 0.01), obesity (AOR = 1.28; 95% CI: 1.10–1.49; *P* < 0.001), waist circumference (AOR = 1.48; 95% CI: 1.24–1.78; *P* < 0.001), TC (≥6.22 mmol/L, AOR = 1.45; 95% CI: 1.19–1.75; *P* < 0.001), TG (≥2.26 mmol/L, AOR = 2.74; 95% CI: 2.39–3.14; *P* < 0.001).

**Conclusions:**

These findings documented that the hyperuricemia is prevalent in the economically developing regions of northwest China. Hyperuricemia is associated with advanced age, male ender and general metabolic and cardiovascular risk factors. Obesity and dyslipidemia increase the risk of hyperuricemia.

## Introduction

Hyperuricemia is an abnormal condition with higher level of uric acid in the blood [1]. In human, if the concentration of serum uric acid (sUA) reaches to 357 μmol/l (6 mg/dL) for women and 416 μmol/l (7.0 mg/dL) for men, hyperuricemia could be diagnosed [2–4]. Hyperuricemia is due to an imbalance of increased production of uric acid or/and decreased excretion of uric acid, which may result in serious complications including gout, tophi, kidney stones, or urate nephropathy even kidney failure [5, 6].

As a cardiovascular risk factor, sUA often accompanies metabolic syndrome, hypertension, diabetes, dyslipidemia and obesity [7]. In developed countries, hyperuricemia was proved to be related to obesity-induced metabolic dysfunction [8] and body weight control can improved lifestyle, dyslipidemia and hyperuricemia and ameliorate metabolic risk factors [9]. Obesity in women and hypertriglyceridemia in men may aggravate hyperuricemia to develop gout [10]. In developing countries, for example, an Indian study reported that the prevalence of hyperuricemia increased significantly in obese population [11]. This is also the case in Bangladesh population [12]. A South African study reported that hyperuricemia was a component and an independent factor of the metabolic syndrome semi urban areas [13]. In Japan and China, a significant association among hypertriglyceridemia, hyperuricemia and obesity was also reported [14]. In Uygur Chinese who living in Xinjiang, Northwest China, an associated among elevated sUA, overweight/obesity and hypercholesteremia has been observed [15–17].

Previous studies have revealed that prevalence of hyperuricemia varies between countries and regions [5, 18–25]. A 21% prevalence of hyperuricemia during 2007–2008 in the US general population [20] and 11.9% in Italian adults (≥18 years) during 2005–2009 were reported [26]. A study estimated the prevalence of hyperuricemia in Chinese adults was 8.4% during 2009–2010 [23], while in Xinjiang (located in northwest China), it was 3.3 to 11.0% in different ethnic groups [16]. A number of studies also observed a trend of increase in hyperuricemia prevalence cross different countries and regions with times [20, 21, 23]. Genetic variations, different life style and some comorbidities e.g. hypertension, obesity, type 2 diabetes may all contribute to the development and onset of hyperuricemia [6, 27–29].

China is the largest developing country, and northwestern China especially Xinjiang is characterized by different ethnic groups and low socioeconomic status with a population of 21.8 million [30]. However, there is no such cross-sectional study designed to investigate the prevalence of hyperuricemia in Xinjiang adults age ≥ 35 years (As we know that hypertension, diabetes, dyslipidemia and other cardiovascular risk factors and atherosclerosis are often well underway before middle age whereas clinical complications are common only after middle age. One aim of the CRS is designed to investigate risk factors for CVDs, so we study the participants aged 35 years and more.) and the association between hyperuricemia and adiposity and dyslipidemia. Therefore, the aim of the current study is to determine the prevalence of hyperuricemia in different ethnic populations and associated risk factors especially adiposity and dyslipidemia in this special region of China.

## Methods

### Study population

**Fig. 1:**
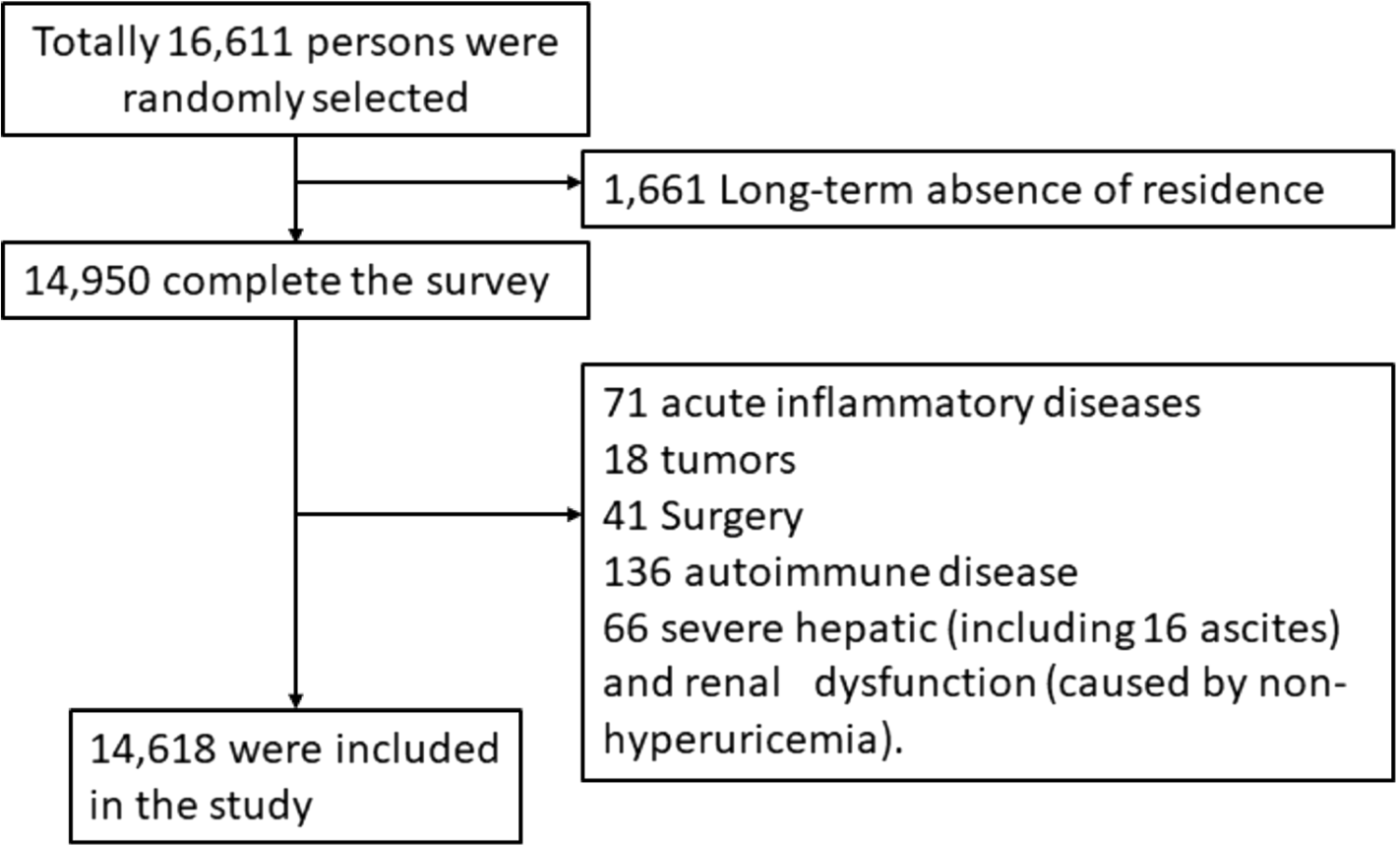
Flow chart of participant recruitment

The Cardiovascular Risk Survey (CRS) is a cross-sectional, observational cohort study designed to investigate the prevalence, incidence, and risk factors for cardiovascular diseases and to determine the genetic and environmental contributions to atherosclerosis, coronary artery disease and cerebral infarction in the Han, Uygur, and Kazakh population in the Xinjiang province of West China and detailed description of study population and methods were described in the previous studies [31, 32]. We used a stratified sampling method to select a representative sample of the general population of Han, Uygur and Kazakh Chinese in this region. The sampling system covered major geographic areas cross Xinjiang. The first level of sampling was stratified by 3 regions including Eastern, Northern and Southern Xinjiang. According to geographical situation, economic status, ethnic distribution, life styles, the overall population size (high/low), proportion of urban population (high/low), and mortality rate (high/low), 7 strata (the second level) were then generated from 7 counties (Urumqi, Kelamayi, Hetian, Zhaosu, Fukang, Tulufan, and Fuhai). The third level of sampling was stratified by urban and rural locations. The fourth level of sampling was stratified by one socioeconomic strata in rural areas and one strata in urban areas [33, 34]. The participants were chosen and classified as urban and rural people based on the government record of registered residence, settlement and location of the people. The inclusion criteria included age ≥ 35 years, local residents in Xinjiang, complete data measurements and informed consents. Participants having one or more of the following conditions were excluded: regular use of cortisone, Cushing syndrome and other endocrinological disease causing body weight gain or loss; excess of alcohol intake and alcohol or drug abuse, other severe illnesses, mental disorders, malignancy, thrombolysis therapy, chronic inflammation, autoimmune disease, hepatic dysfunction, ascites, chronic renal failure or serum creatinine level > 2.5 mg/dL (not caused by hyperuricemia) and other serious diseases. In total, 1993 subjects were excluded from the study, and 14,618 participants from these seven areas (with a response rate of 88.8%) were included in this study (Fig. 1).

### Anthropometric measurements

An interview-based questionnaire was used to gather information on the following: general health status, seafood or internal organs intake, history of diseases, etc. It was designed in the Chinese language but translated into Uygur or Kazakh language for those who didn’t understand Chinese. The questionnaire collected information including demographic, socioeconomic status and medical history. We also performed a physical examination including the measurements of height, body weight, systolic blood pressure (SBP) and diastolic blood pressure (DBP). Body weight was measured to the nearest 0.1 kg in light clothing with a mechanical scale. Height was measured to the nearest 0.1 cm without shoes, with a commercial stadiometer (HW-900B OMRON, Japan). For those with physical deformity, Demi-span was measured, and height is then calculated from a standard formula. With the subjects standing and breathing normally, using a measuring tape parallel to the floor, waist circumference (WC) was measured at midpoint between the last palpable rib and the anterior superior iliac spine and the hip circumference (HC) was measured at the outermost points of the greater trochanters [35]. The ratio of WC to HC (WHR) was calculated. Blood pressure was measured three times with a 5 min interval, by tail cuff method.

### Blood collection and laboratory test

Venous blood samples were drawn at overnight fasting condition (12 h). All routine full blood examination and blood biochemistry were performed using the commercially available automated platform, in the Central Laboratory of the First Affiliated Hospital of Xinjiang Medical University. These tests included sUA, total cholesterol (TC), triglyceride (TG), low- or high-density lipoprotein-cholesterol (LDL-C, HDL-C), fasting blood glucose (FBG), blood urea nitrogen (BUN) and serum creatine (with eGFR calculated).

### Definition of cardiovascular risk factors

Hypertension was defined as history of hypertension and/or repeated systemic blood pressure measurements exceeding 140/90 mmHg [36]. Subjects with body mass index (BMI) between 25 and 28 kg/m^2^ were defined as overweight, and subjects with BMI ≥ 28 kg/m^2^ were defined as obese [37, 38]. Participants with a WC ≥85 cm for men and WC ≥80 cm for women was defined as a risk factor [38]. Diabetes was defined as history or presence of diabetes and/or a fasting plasma glucose level ≥ 7.0 mmol/L (126 mg/dL) or a random glucose value ≥11.1 mmol/L (200 mg/dL) or a 2-h plasma glucose ≥11.1 mmol/L during an oral glucose tolerance test (OGTT) plus signs or symptoms of diabetes. If there is no signs or symptoms of diabetes, the glucose level should be examined on another day [39]. Concentrations of glucose ≥6.1 mmol/L, TC ≥6.22 mmol/L, TG ≥2.26 mmol/L, LDL-C ≥ 4.14 mmol/L, and HDLC < 1.04 mmol/L were defined as hyperglycemia, hypercholesterolemia, hypertriglyceridemia, hyper-LDL-C or hypo-HDL-C, respectively [40].

### Statistical analysis

The collected data were independently recorded and verified by two staff members using EpiData 3.02 software (EpiData Association, Odense, Denmark). Statistics were analyzed using SPSS21.0 (SPSS, Inc., Chicago, IL, USA). Continuous variables were expressed as mean ± standard deviation (SD). Categorical variables were compared using the Chi-square or Fisher’s test and expressed as percentage. Changes of general and clinical characteristics and serum levels of biochemical parameters in the participants according to Quintiles of sUA were analyzed. To examine the variability between observers, we conducted inter- and intra-observation in some anthropometric parameters. Coefficients of inter- and intra-observer variability were calculated using a paired t-test. We used logistic regression models to analyze association between hyperuricemia and other potential metabolic and cardiovascular risk factors in two steps. We first applied a univariate logistic regression model to evaluate association among hyperuricemia and the variables listed in Table 1. We then performed a multivariate logistic regression model to assess the association between hyperuricemia and possible confounding factors used in the univariate analysis. Crude odds ratios (COR) and adjusted odds ratios (AOR) were calculated along with 95% confidence intervals (CIs). Statistical significance was set at *P* < 0.05.

**Table 1:** Comparison of general characteristics and serum levels of biochemical parameters between non-hyperuricemia and hyperuricemia populations

	All participants		Non- hyperuricemia		Hyperuricemia		*P* value
	*n* or Mean	% or SD	*n* or Mean	% or SD	*n* or Mean	% or SD	
Age (years)	50.8	12.6	50.5	12.5	53.9	13.2	< 0.001
Men, n (%)	6819	46.6	6011	45.2	808	60.7	< 0.001
Height (cm)	162.0	8.7	161.7	8.6	164.7	9.2	< 0.001
Body weight (kg)	67.8	13.3	67.2	13.1	74.3	13.6	< 0.001
BMI (kg/m^2^)	25.8	4.2	25.6	4.2	27.3	4.1	< 0.001
SBP (mmHg)	134	22	133.9	22.2	140.1	21.9	< 0.001
DBP (mmHg)	84	16	83.8	16.8	89.1	17.3	< 0.001
Waist circumference (cm)	87.6	11.9	87.1	11.8	93.2	11.2	< 0.001
Hip circumference (cm)	97.5	9.1	97.2	9.1	99.6	8.7	< 0.001
Waist to hip ratio	0.90	0.09	0.90	0.09	0.94	0.08	< 0.001
Alcohol intake, n (%)	2151	14.7	1799	13.5	352	26.4	< 0.001
Smoking, n (%)	4169	28.5	3640	27.4	529	39.7	< 0.001
Seafood or/and internal organs intake	976	6.7	860	6.5	116	8.7	< 0.001
Overweight, n (%)	5351	36.6	4786	36.0	565	42.4	< 0.001
Obesity, n (%)	3881	6.5	3387	25.5	494	37.1	< 0.001
Hypertension, n (%)	5731	39.2	5069	38.2	662	49.7	< 0.001
Diabetes, n (%)	915	6.3	799	6.0	116	8.7	< 0.001
Hypertriglyceridemia	4161	28.5	3412	25.7	749	56.3	< 0.001
Hypercholesterolemia	3834	26.2	3249	24.5	585	44.0	< 0.001
sUA (umol/l)	274.8	85.2	257.4	66.7	442.1	58.3	< 0.001
FBG (mmol/L)	5.2	1.7	5.1	1.7	5.4	1.6	< 0.001
TC (mmol/L)	1.6	1.3	1.5	1.2	2.3	1.8	< 0.001
TG (mmol/L)	4.6	1.1	4.6	1.1	5.1	1.2	< 0.001
HDL–c (mmol/L)	1.3	0.5	1.3	0.5	1.2	0.5	< 0.001
LDL–c (mmol/L)	2.9	0.9	2.9	0.9	2.9	0.9	0.47
Serum creatine (mmol/L)	72.9	25.9	71.3	24.8	88.7	30.9	< 0.001
eGFR (ml/min/1.73 m^2^)	84.3	27.8	85.3	27.9	69.2	20.2	< 0.001

*BMI* body mass index**,***SBP* systolic pressure**,***DBP* diastolic blood pressure**,***sUA* serum urate**,***FBG* fasting blood glucose**,***TG* total triglyceride**,***TC* total cholesterol**,***HDL–C* high density lipoprotein–cholesterol**,***LDL–C* low density lipoprotein–cholesterol**,***eGFR* estimated glomerular filtration rate

## Results

### General characteristics and biochemical parameters of study population

General characteristics and biochemical parameters are shown in Table 1. All of the study population were stratified into two groups according to presence of hyperuricemia. Those hyperuricemia participants tended to be elder, higher blood pressure, greater BMI, WC and HC (all *P* < 0.001). In addition, they tended to smoke more, and had more alcohol intake and seafood or internal organs intake. More hyperuricemia participants combined with metabolic diseases including hypertension, diabetes and lipid disorders than those without hyperuricemia (*P* < 0.001).

Of note, mean sUA in participants without or with hyperuricemia was 257.4 ± 66.7 and 442.1 ± 58.3 umol/l respectively (*P* < 0.001). Hyperuricemia participants had higher levels of FBG, TC, TG, serum creatinine and eGFR, and lower level of HDL-c compared with non-hyperuricemia participants (All *P* < 0.001).

### Comparison of characteristic and serum biochemical parameter levels of the participants according to quintiles of sUA

Table 2 presents changes of characteristic and serum levels of biochemical parameters in participants according to Quintiles of sUA. Participants with higher levels of sUA were elder males and have higher BMI and blood pressure compared to those with lower levels of sUA (both *P* < 0.001). Among them, more people tended to smoke and drink (*P* < 0.001). Participants with diabetes, hypertension or hypertriglyceridemia and higher eGFR, FBG, TG, TC were associated with higher levels of sUA (all *P* < 0.001).

**Table 2:** Comparison of general characteristics and serum levels of biochemical parameters in study participants according to Quintiles of serum levels of uric acid

Variables	1st Quartile < 213 umol/l (*n =* 4014)		2nd Quartile 214–262 umol/l (*n =* 3515)		3rd Quartile 263–326 umol/l (*n* = 3560)		4th Quartile > 327 umol/l (*n* = 3529)		*P* Value
	*n* or Mean	% or SD	*n* or Mean	% or SD	*n* or Mean	% or SD	*n* or Mean	% or SD	
Age (years)	48.4	12.0	50.5	12.3	52.1	12.6	52.62	13.1	< 0.001
Gender (male), n and %	1035	25.8	1275	36.3	1909	53.6	2600	73.7	< 0.001
History of smoking, n and %	654	16.3	778	22.1	1170	32.9	1567	44.4	< 0.001
Alcohol intake, n and %	232	5.8	299	8.5	582	16.4	1038	29.4	< 0.001
BMI (kg/m^2^)	24.9	4.2	25.3	4.2	26.1	4.3	26.91	3.9	< 0.001
SBP (mmHg)	131.4	22.5	132.8	21.9	135.7	22.2	138.2	21.5	< 0.001
DBP (mmHg)	81.2	16.6	83.2	16.6	85.3	16.9	87.9	16.8	< 0.001
Hypertension, n and %	1303	32.5	1289	36.7	1509	42.4	1600	45.3	< 0.001
BUN (mmol/L)	4.6	1.5	4.8	1.5	5.1	1.5	5.4	1.7	< 0.001
Creatine (mmol/L)	61.5	24.0	68.7	23.6	75.3	23.0	86.2	26.4	< 0.001
eGFR (ml/min/1.73 m^2^)	97.2	34.1	86.4	25.9	80.5	22.9	73.1	20.8	< 0.001
FBG (mmol/L)	5.0	1.9	5.1	1.7	5.2	1.5	5.3	1.6	< 0.001
Diabetes	184	4.6	185	5.3	230	6.5	260	7.4	< 0.001
TG (mmol/L)	1.2	0.9	1.3	1.0	1.6	1.3	2.1	1.6	< 0.001
TC (mmol/L)	4.2	1.1	4.5	1.1	4.8	1.1	5.0	1.1	< 0.001

*BMI* body mass index**,***SBP* systolic blood pressure**,***DBP* diastolic blood pressure**,***sUA* serum uric acid**,***FBG* fasting blood glucose**,***TG* total triglyceride**,***TC* total cholesterol**,***HDL–C* high density lipoprotein–cholesterol**,***LDL–C* low density lipoprotein–cholesterol

### Variability between observers

Table 3 shows variability between observers. The coefficients of variability in both inter- and intra-observer were small for both height, weight measurements, et al. Regarding blood pressure, the variation of intra-observer was smaller than that of inter-observer. All data were within the 95% confidence interval. There was no systematic error between inter- and intra-observations (all *P* > 0.05). These results indicate that this method is reliable.

**Table 3:** Variability of inter-observation and intra-observation (*n* = 50)

	Inter-observer				Intra-observer			
	Error (%)		Coefficient		Error (%)		Coefficient	
	Mean	SD	Mean	SD	Mean	SD	Mean	SD
Height (cm)	−1.3	68.7	0	11	2.9	15.8	0.1	2.8
Body weight (kg)	0.4	70	0.3	21	10.7	25.8	0.1	0.5
Waist circumference (cm)	0.5	70.2	0	34	3.5	21.1	0.1	0.5
Hip circumference (cm)	0.8	69.9	−1	27	27.1	42.0	0.9	2.5
SBP (mmHg)	0	70	0	18	16.7	30.6	0.2	0.5
DBP (mmHg)	0.2	69.4	0.2	13	12.6	27.1	0.2	0.7

*SBP* systolic blood pressure, *DBP* diastolic blood pressure

### Changes of characteristic and serum levels of biochemical parameters of the participants according to ethnicities

Table 4 presents changes of characteristic and serum levels of biochemical parameters of the participants according to ethnicities. There were variations in the distributions of age, BMI, SBP,

**Table 4:** Comparison of clinical characteristics and levels of serum biochemical parameters among three ethnic groups

Ethnicity	Han (*n* = 5757)		Uygur (*n* = 4767)		Kazakh (*n* = 4094)	
	*n* or Mean	% or SD	*n* or Mean	% or SD	*n* or Mean	% or SD
Age (years)	52.5	12.7	50.7*	13.0	48.6*†	11.7
BMI (kg/m^2^)	25.1	3.5	25.8*	4.4	26.6*†	4.8
SBP (mmHg)	133	20	131*	21	140*†	25
DBP (mmHg)	85	16	80*	15	88*†	20
FBG (mmol/L)	5.3	1.8	4.9*	1.7	5.1*†	1.5
Alcohol intake, n and %	1098	19.1	466*	9.8	587 (3)*†	14.
Smoking, n and %	1767	30.7	845*	17.7	203*†	5.0
Coffee/tea consumption, n and %						
Never	2245	39.0	845*	17.7	203*†	5.0
Occasionally	1783	31.0	1119*	23.5	120*†	2.9
Often	1729	30.0	2803*	58.8	3771*†	92.1
Seafood or/and internal organs intake, n and %	346	6.0	553	11.6	77	1.9
TG (mmol/L)	1.7	1.5	1.6*	1.2	1.2*/†	0.9
TC (mmol/L)	4.7	1.1	4.4*	1.1	4.8*†	1.2
HDL-C (mmol/L)	1.3	0.5	1.3	0.5	1.3*†	0.4
LDL-C (mmol/L)	2.9	0.9	2.9	0.9	2.9	0.9
BUN (mmol/L)	4.9	1.5	5.2*	1.7	4.7*†	1.5
Creatine (umol/L)	75.8	26.1	71.4*	29.7	70.5*	19.8
_S_UA (umol/L)	306.2	86.9	249.4*	76.1	259.8*†	78.7

*BMI* body mass index, *SBP* systolic pressure, *DBP* diastolic blood pressure, *FBG* fasting blood glucose, *TG* total triglyceride, *TC* total cholesterol, *HDL–C* high density lipoprotein–cholesterol, *LDL–C* low density lipoprotein–cholesterol, *sUA* serum urate acid. P* < 0.05: compared to Han ethnic patients. P † < 0.05: compared to Uygur ethnic patients

DBP, alcohol intake, smoking and coffee/tea consumption. Han Chinese had lower BMI, but tended to have more drinking, smoking and coffee/tea consumption and highest mean level of sUA to other ethnic groups (*P* < 0.05 respectively). Uygur Chinese had lower levels of SBP, DBP, FBG and TC compared to other ethnic groups and had lowest mean level of sUA (*P* < 0.05 respectively). Kazakhs were more likely to have higher BMI, SBP, DBP, TC and HDL-c, while the level of sUA was less than Han Chinese but higher than Uygur Chinese (both *P* < 0.05).

### Prevalence of hyperuricemia and mean sUA estimations in Xinjiang in 2008–2012

The overall prevalence of hyperuricemia in Xinjiang adults aged ≥35 years during 2008–2012 was 9.1% (95% CI: 8.6–9.6), which was higher in men than in women (11.1% vs. 6.2%) and increased with aging (Table 5).

**Table 5:** Comparison of prevalence of hyperuricemia in participants under classifications of age, gender, residence, eGFR and ethnicity

	Study population (*n*)	Hyperuricemia (*n*)	Prevalence (%)	95% CI	*P*
Age (years), *n* and %					< 0.001
35–44	5425	397	7.3	6.6–8	
45–54	3759	328	8.7	7.8–9.6	
55–64	2932	270	9.2	8.2–10.3	
65–74	1989	266	13.4	11.9–14.9	
75–101	513	70	13.6	10.7–16.6	
Gender, *n* and %					< 0.001
Men	6819	808	11.8	11.1–12.6	
Women	7799	523	6.7	6.2–7.3	
Residence, *n* and %					< 0.001
Rural	7974	967	12.1	11.4–12.8	
Urban	6644	364	5.5	4.9–6	
eGFR (ml/min/1.73 m^2^), n and %					< 0.001
≥ 90	5007	151	3	2.5–3.5	
60–89	6787	747	11	10.3–11.8	
30–59	2217	404	18.2	16.6–19.8	
15–29	95	23	24.2	15.6–32.8	
< 15	512	6	1.2	0.2–2.1	
Ethnicity, *n* and %					< 0.001
Han	5757	884	15.4	14.4–16.3	
Uygur	4767	220	4.6	4–5.2	
Kazakh	4094	227	5.5	4.8–6.2	
Total	14,618	1331	9.1	8.6–9.6	

*eGFR* estimated glomerular filtration rate

The prevalence of hyperuricemia was higher in rural area than in urban area (12.1% vs. 5.5%, *P* < 0.001) and it increased with advancing chronic kidney disease stage based on eGFR (> 15 umol/min/1.73m^2^). The ethnicity specific prevalence rate of hyperuricemia was much higher in Han than Uygur and Kazakh populations ((*P* < 0.001, Table 5).

### Association between prevalence of hyperuricemia and adiposity and dyslipidemia

A univariate logistic regression showed that hyperuricemia was associated with age, gender (male), ethnicity, smoking, drinking, hypertension, diabetes, overweight/obesity, WC, WHR, TC (≥6.22 mmol/L), TG (≥2.26 mmol/L) and LDL-C (Table 6).

**Table 6:** Univariate and multivariate logistic analyses for the effects of adiposity, lipid profile and characteristics of subjects on the risk of hyperuricemia

	*Univariate logistic analysis*			*Multivariate logistic analysis*		
	COR	95% CI	*P* Value	AOR	95% CI	*P* Value
Age, years						
35–44	Ref			Ref		
45–54	1.21	1.04–1.41	0.01	1.06	0.90–1.24	0.51
55–64	1.28	1.09–1.51	< 0.001	1.16	0.97–1.38	0.11
65–74	1.96	1.66–2.31	< 0.001	1.61	1.34–1.94	< 0.001
75- and older	2.00	1.52–2.63	< 0.001	1.71	1.27–2.29	< 0.001
Gender						
Women	Ref			Ref		
Men	1.87	1.67–2.10	< 0.001	1.45	1.24–1.68	< 0.001
Ethnic						
Uygur	Ref			Ref		
Han	3.75	3.22–4.37	< 0.001	3.81	3.24–4.47	< 0.001
Kazak	1.21	1.01–1.47	0.047	1.31	1.07–1.61	< 0.001
Smoking						
No	Ref			Ref		
Yes	1.75	1.56–1.96	< 0.001	1.01	0.87–1.19	0.86
Drinking						
No	Ref			Ref		
Yes	2.30	2.01–2.62	< 0.001	1.36	1.16–1.61	< 0.001
Hypertension						
No	Ref			Ref		
Yes	1.59	1.42–1.78	< 0.001	1.12	0.99–1.27	0.08
Diabetes						
No	Ref			Ref		
Yes	1.59	1.30–1.96	< 0.001	0.84	0.68–1.05	0.13
Overweight (BMI ≥25 and < 28 kg/m^2^)						
No	Ref			Ref		
Yes	2.18	1.93–2.46	< 0.001	1.25	1.06–1.48	0.01
Obesity (BMI ≥28 kg/m^2^)						
No	Ref			Ref		
Yes	1.72	1.53–1.94	< 0.001	1.28	1.10–1.49	< 0.001
WC (men ≥90 cm; women ≥85 cm)						
No	Ref			Ref		
Yes	2.33	2.07–2.64	< 0.001	1.48	1.24–1.78	< 0.001
WHR (men > 0.90; women > 0.85)						
No	Ref			Ref		
Yes	2.07	1.82–2.36	< 0.001	1.17	0.99–1.38	0.07
TC ≥6.22 mmol/L						
No	Ref			Ref		
Yes	1.97	1.65–2.35	< 0.001	1.45	1.19–1.75	< 0.001
TG ≥2.26 mmol/L)						
No	Ref			Ref		
Yes	3.79	3.35–4.28	< 0.001	2.74	2.39–3.14	< 0.001
LDL–C ≥ 4.14 mmol/L						
No	Ref			Ref		
Yes	0.83	0.67–1.04	0.10	0.86	0.68–1.09	0.21
HDL–C < 1.04 mmol/L						
No	Ref			Ref		
Yes	1.20	1.06–1.35	< 0.001	0.88	0.78–1.00	0.05

*BMI* body mass index**,***WHR* weight to hip ratio**,***TC* total cholesterol**,***TG* total triglyceride**,***HDL–C* high density lipoprotein–cholesterol**,***LDL–C* low density lipoprotein–cholesterol**,***COR* crude odds ratio**,***AOR* adjusted odds ratio

Multivariate logistic regression analysis revealed that age, gender, ethnicity, drinking, obesity, waist circumference, TG (≥2.26 mmol/L), TC (≥6.22 mmol/L) are major risk factors for hyperuricemia. Compared to the 35–44-year age group [adjusted odds ratio (AOR) = 1], the risk of hyperuricemia was 1.61-fold in the 65–74-year age group (AOR = 1.61, 95% CI: 1.34–1.91; *P* < 0.001), and 1.71-fold in the 75- and older age group (AOR = 1.71, 95% CI: 1.27–2.29; *P* < 0.001). There was a 1.45-fold higher risk of hyperuricemia in men (AOR = 1.45, 95% CI: 1.24–1.68; *P* < 0.001) compared to women. Further, the risk of hyperuricemia increased significantly with drinking (AOR = 1.36; 95% CI: 1.16–1.61; *P* < 0.001), overweight (AOR = 1.25; 95% CI: 1.06–1.48; *P* = 0.01), obesity (AOR = 1.28; 95% CI: 1.10–1.49; *P* < 0.001), waist circumference (AOR = 1.48; 95% CI: 1.24–1.78; *P* < 0.001), TC (≥6.22 mmol/L, AOR = 1.45; 95% CI: 1.19–1.75; *P* < 0.001), TG (≥2.26 mmol/L, AOR = 2.74; 95% CI: 2.39–3.14; *P* < 0.001, Table 6).

## Discussion

In the current study, we found that the prevalence of hyperuricemia in Xinjiang was 9.1% during the period of 2008 to 2012. The risk of hyperuricemia in northwest China increased significantly with aging, different ethnicities, adiposity and dyslipidemia. People with more consumption of alcohol, overweight, obesity, higher WC, TC and TG tended to have hyperuricemia. Compared to Uygur and Kazakh Chinese, Han Chinese were more predisposed to hyperuricemia.

In our study we observed a 9.1% overall prevalence of hyperuricemia in Xinjiang which is higher than that in US general population (3.2%) during the period of 2007–2008 [20] and that in other region of Chinese adults (8.4%) during the period of 2009–2010 [23]. This may be attributed to aging [41], increased prevalence of overweight and obesity [17, 23], lifestyle changes, especially higher energy intake [42], increased consumption of foods rich in purines [43], diet soft drink [44], consumption of alcohol [24, 45] and physical inactivity [46]. In Xinjiang, Uygur, Han and Kazakh Chinese consist of the majority of population and account for 46, 40 and 7%, respectively [47]. We found that Han Chinese suffered more hyperuricemia than Uygur and Kazakh Chinese. The genetical background may have a potential influence in the prevalence of hyperuricemia among these different ethnic groups except lifestyle, a major factor. For example, previous studies reported that gene polymorphism in Uygur Chinese may be correlated with hyperuricemia such as ApoE E4, IL-8, IL-1RL1, IL-18 and SLC17A1 genes [48–50]. Hyperuricemia is a complicated metabolic disease with many risk factors. Among them, clarification of causal and protective genes would be very important in understanding the pathogenesis of hyperuricemia. To find ethnic difference and further exploring genetic variations would be a useful procedure for relevant biologic or metabolic evidence.

Weight gain was considered to be one strong risk factor for hyperuricemia. In consistent with previous reports [51–53], we found that risk of hyperuricemia was higher under the condition of obesity and overweight. In recent 40 years, along with rapid economic development in China, lifestyle has changed significantly. The prevalences of overweight and obesity increased from 22.8 to 30.1% and from 7.1 to 11.9% during the period of 2002–2012, respectively [54]. We also observed that participants with normal sUA levels had lower BMI than hyperuricemia participants. In Xinjiang, the prevalence of obesity ranged from 11.5 to 17.9%, in particular, the central obesity ranged from 53.9 to 59.9%, it is higher than the national levels [55]. And higher prevalence of obesity may be one explanation of higher prevalence of hyperuricemia in Xinjiang than national prevalence of hyperuricemia in China, while normal or lower BMI may be a protective factor.

Higher prevalence of hyperuricemia was also found to be correlated with dyslipidemia including higher TG and TC levels in adults aged 35 years and older adults. Hypertriglyceridemia is the most common risk factor for hyperuricemia. Peng et al. reported that higher TG, TC and LDL-c correlated significantly with elevated sUA, while HDL-C levels were negatively related to sUA levels [56]. Rathmann et al. showed that elevated TG level was an independent risk factor in males with hyperuricemia [57]. Takahashi et al. found that serum concentration of TG was associated with gout. Our results were consistent with those previous studies and we found higher levels of TG, TC, LDL-C and lower levels of HDL-C in hyperuricemia subjects although the association between higher levels of serum LDL-C and lower levels of HDL-C and higher prevalence of hyperuricemia remained elusive (with no significant difference in multiple regression analysis). According to these data, hyperuricemia may predict an unfavorable lipid profile, especially higher values of TG and TC levels. It needs more comprehensive intervention on those dyslipidemia patients combined with hyperuricemia.

Increase of seafood or internal organs intake was often considered as a cause of elevated sUA. It was reported that higher levels of consumption of seafood and dairy products were associated with increase in risk of gout [27]. The adverse effect of seafood or internal organs intake may be worse in patients with gout. Purines are broken down into uric acid. Purine intake had been proved to be associated with hyperuricemia and increased risk of incident gout [27]. A diet rich in purines from certain sources including seafood, meat and internal organs can raise sUA, which sometimes leads to gout [6]. In eastern coastal regions, such as Han Chinese in Qingdao and Hangzhou who had higher prevalence of hyperuricemia than Han Chinese in Xinjiang (25.3% vs. 16.9% vs. 15.4%) [24]. However, in our study, we found that seafood or/and internal organs intake was not an independent risk factor for development of hyperuricemia although hyperuricemia subjects consumed more seafood or/and internal organs. The exact reason for the discrepancy is unclear, more consumption of meat and dairy foods by the residents in Xinjiang than other regions of mainland of China may be a potential factor. Compared to Han and Uygur Chinese, Kazakh consumed more dairy products but the prevalence was not the lowest, and one of the possible explanation may be along with higher consumption of meat and less vegetables and fruits [58].

We also found ethnic difference of lipid levels among Han, Uygur and Kazakh populations. Regarding TG and TC levels, Han Chinese were significantly higher than Uygur and Kazakh Chinese. Lifestyle could affect TC, HDL-C, LDL-C, and TG levels in patients with dyslipidemia [59, 60]. Evidence showed that dyslipidemia contributed to both the onset and progression of atherosclerosis while the evidence-based treatment could decrease cardiovascular events [61]. It had been proven that the combination of a lipid-lowering diet and nutraceutical supplements could reduce LDL-C and increase HDL-C [62]. A recent review by Scicchitano P et al. pointed that natural nutraceuticals including niacin, omega-3 fatty acids, red yeast rice, fiber, plant sterols, and alpha linolenic acid (ALA) showed substantial effects in reductions in both serum lipid profiles [63]. Different ethnics have different social environment and diets. In Xinjiang, Uygur and Kazakh populations have a unique lifestyle. Kazakh Chinese consume more salt, wheaten food, meat and milk [64] while Uygur Chinese has a relatively special diet, such as high-sugar, high-fat, and low-dietary fiber diet [65]. There is difference in the lipid profile in different ethnic groups in Xinjiang [66]. And physical activity has important effects in dyslipidemia improvement [67, 68]. Beneficial effects of physical activity even intensities of exercise all lower cholesterol levels which may associated with body fat loss [69]. Base on the beneficial effects of physical activity, intensive aerobic exercise has been recommended for patients with dyslipidemia. For example, increasing physical activity to 30 min/day 5 times weekly and prolonging moderate-intensity aerobic exercise at 70–80% heart rate (HR) _reserve_, progressing to 85% HR_max_ could help to reduce LDL-C and TG and increase HDL-C [70]. However, for the nature of cross-sectional study, nutraceuticals effects on dyslipidemia were not included in the present study and more efforts may be made to investigate influence of diet habits and lifestyle changes in this special area.

Some limitations of the study deserved to be mentioned. First, cross-sectional nature of this study determines that all participants are not followed up, thus we don’t know the impact of changes in lifestyle on the prevalence of hyperuricemia. Second, although populations of Han, Uygur and Kazakh possessed majority population of Xinjiang, we didn’t recruit other 10 ethnicities accounting for 7% population of Xinjiang. Third, gout is an inflammatory arthritis associated with hyperuricemia and the epidemic status of gout should be studied along with hyperuricemia, while our study didn’t include this at the initial design. Fourth, due to variations in educational level and language barrier in this multiethnic region, information provided by participants may be incomplete. This collection of information may bring bias when performing data analysis. Despite these, our study provides an overall picture and latest information for the prevalence of hyperuricemia in three different ethic population and associated risk factors in Xinjiang.

## Conclusions

These findings documented that the hyperuricemia is prevalent in the economically developing regions of northwest China. Hyperuricemia is associated with advanced age, male ender and general metabolic and cardiovascular risk factors. Obesity and dyslipidemia increase the risk of hyperuricemia. There is an urgent need to effectively manage these factors for preventing further increases in the burden of hyperuricemia and it also needs more comprehensive intervention on those individuals with metabolic disorders combined with hyperuricemia.

## Data Availability

The datasets used and/or analyzed in the current study are available from the corresponding authors upon reasonable request.

## Abbreviations

BMI: Body mass index
BUN: Blood urea nitrogen
DBP: Diastolic blood pressure
eGFR: Estimated glomerular filtration
FBG: Fasting blood glucose
HDL-C: High density lipoprotein cholesterol
LDL-C: Low density lipoprotein cholesterol
SBP: Systolic blood pressure
sUA: Serum uric acid
TG: Triglyceride
WC: Waist circumference
WHR: Waist-to-hip ratio
